# Trismus Resulting from Infantile Hemangioma of the Parotid: A Rare Case Report

**Published:** 2015-12-10

**Authors:** E Zarepur, M Moghimi

**Affiliations:** 1 Medical Student, Student Research Committee, ShahidSadoughi university of medical sciences, Yazd, Iran; 2 Assistant Professor, Department of Pathology, Shahidsadoughi University of Medical Sciences, Yazd,Iran

**Keywords:** Parotid, hemangioma, trismus, vascular abnormality

## Abstract

Vascular abnormalities are characterized by increasing number of vessels. Salivary gland tumors are uncommon and their overall incidence is about 3 per 100000 per year. Salivary gland hemangioma makes up 1 % of all salivary gland tumors. Trismus resulting from parotid hemangioma is so rare. The patient was a 6-month-old boy with a huge lesion in his right parotid who referred to Shahid sadoughi hospital of Yazd, Iran. The lesion appeared at 4 months of age and had rapid growth and was suspected as hemangioma after clinical examination and patient had trismus. The lesion was excised without any complications. Five months after surgery, area of the lesion appeared normal. Parotid hemangioma has low potential to turn into malignant form but early detection and biopsy are necessary for decreasing complications. Removal of the mass was the best treatment for the patients with large or complicated hemangioma; however, it should be performed with caution because the tissues may bleed profusely. Patients may have long term survival after surgery.

## Introduction

Hemangioam is a kind of endothelial malformation and can cause significant morbidity and mortality in children and adults. The term hemangioma is applied to describe a benign vascular (with growth phase) and self-limited tumor. A hemangioma can occur anywhere on the body. Mechanisms of controlling the growth of hemangiomas are not clearly understood. Infantile hemangiomas are characterized by proliferation of vessels within the first year of life. Parotid hemangioma is a benign rare lesion (1) and is more common in females (2).

In most cases, the cause of hemangioma is unknown but low birth weight probably is the major risk factor and most of hemangiomas are asymptomatic (3).The duration of the growth phase can be variable, but most hemangiomas reach their maximal size within the first 9 months of life(4).

Treatment of a hemangioma isn't usually needed but the care of children with hemangioma is the most important part of the treatment .In this report, a case of parotid hemangioma was described and as far as we know it is the first case reported in Iran and a rare case in the world.

## Case report

A 6 month old male infant presented with a right-sided hemihypertrophy of the face in the preauricular region. He was referred to Shahid Sadoughi Hospital of Yazd, Iran, for evaluation and treatment of the swelling that it was appeared initially as a bright red swelling with rapid growth. The lesion began at 4 months of age with rapid growth. The size of tumor was 7×7×3.5.It was a mobile, soft, and bright red swelling in the region of parotid gland.

**Figure 1:**
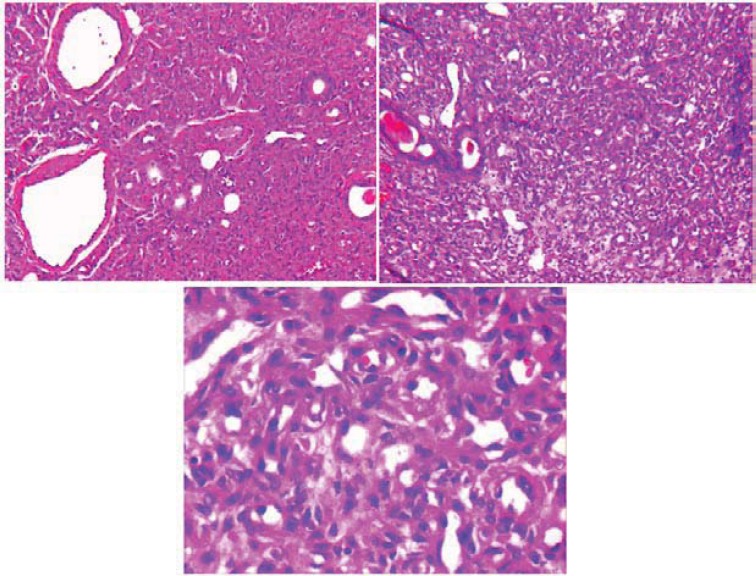
Photomicrograph showing a lobular proliferation of endothelial cells forming small lumens within salivary gland (H&E

Swelling's size with changing the position of the head did not change. No evidence of facial weakness or cervical lymphadenopathy was presented, however he had some degree of trismus. The patient was taken no medications for this condition before his treatment. When the mass failed to respond to a 3-week course of steroid treatment (and trismus continued), the patient underwent surgical resection of the parotid mass. The vascular lesion was excised and submitted for histopathological examination. Histologic examination of hematoxylin and eosin stained slides showed dilation, proliferation of vessels and A large number of thin-walled capillary vascular spaces containing red blood cells. The residual salivary gland within tumor was also seen in Fig.1. After clinical and physical examinations and pathological evaluation in the pathology department, the case was diagnosed as the capillary hemangioma. The lesion was excised without any complications. 18 months after surgery, the area of the lesion appeared normal, the area had healed and no complication was observed 6 months after surgery.

## Discussion

Hemangiomas are vascular abnormalities and characterized by increasing number of vessels. The salivary glands are responsible for making saliva and enzymes and releasing them into the mouth. Hemangiomas are classified as major (parotid, submandibular, and sublingual) and minor. The salivary gland tumors are uncommon and allocated 2-4% of head and neck neoplasms to themselves. Most (70%) salivary gland tumors are in the parotid gland(5). Most of tumors of the parotid gland are benign. They are more common among white females than white males(2). Infantile hemangioma typically grows fast and turn into red or bruise like mass and may become very large, causing severe facial deformity(4). To our knowledge, the occurrence of trismus following parotid hemangioma is so rare. Trismus or lockjaw is tonic contraction of the muscles of mastication. In this case, the growth was prominent and he had moderate difficulties of sucking. Although, the mangioma is the most common soft tissue tumor of infancy but it is relatively rare in the parotid gland. Salivary gland hemangioma makes up 1 % of all salivary gland tumors (6-8).

Common differential diagnosis hemihypertrophy of the face are hemangioma, malignant neoplasm, and abscess (9). If biopsy specimen is small the pathologist should be aware of the possibility of a primary salivary gland angiosarcoma to prevent a misdiagnosis (10). Hemangioma usually diagnosed as a bruis; nevertheless, It's diagnose is possible with further growth. Physical examination is usually diagnostic in typical cases. Imaging modalities such as sonography and MRI can help physicians in diagnosing hemangioma. Hemangiomas are usually hypoechoic at ultrasonography and display abnormal flow at doppler ultrasonography. A color Doppler sonogram usually shows a hypervascular mass (tortuous arterial and venous). MRI also is a good choice (information on the size and deep extent of the tumor)(11). In this case doppler ultrasonography was performed for diagnosing this disease.

In some cases, hemangiomas can be potentially life-threatening. Available treatments such as corticosteroids have variable responses and side effects (12). Treating hemangiomas is a challenge because of the lesion's expansion and resistance to treatment. The parotid requires special consideration due to its susceptibility to consequent bleeding, facsial paralysis, and cosmetic issues in most cases.

Hemangiomas can be deep, superficial or segmental (segmental hemangiomas have more complications than localized hemangiomas). Therefore various treatment modalities have been attempted and consequently, surgical resection is chosen as a safe procedure with lower relapse. Surgical excision during the proliferative phase should be avoided due to risk of major blood loss and injury to the facial nerve. Recently, bleomycin and oral propranolol have been introduced but no effective medical treatment has been reported for children with large and forming hemangiomas of the parotid gland (1, 13). 

In addition, propranolol and corticosteroids are safe drugs that can be used (especially in capillary hemangioma). Furthermore, in some cases resistance to propranolol in at least 9% of thepatients is seen. Despite the efficacy of propranolol, patients must be monitored in life-threatening hemangiomas like airway hemanigiomas(12). As for other infantile hemangiomas, propranolol proved to be an effective, safe, and well-tolerated treatment for parotid hemangioma (14, 15). Corticostroid was selected as the first choice of therapy because it was the most effective and available medicine for treating hemangioma in hemangiomas. Propranolol was not chosen as treatment in this study because of large size and rapid growth of the tumor as well as presence of trismus and feeding problems.

 In this case, the lesion wasn’t a segmental hemangioma. Since the first choice was medical treatment but it didn't work. So, it was performed surgical resection. 

## Conclusion

This article demonstrated that hemangiomas in the parotid gland may be more resistant to therapy with corticosteroids so the surgical procedure can be a safe treatment. Despite different recommended modalities in managing parotid hemangiomas, in cases of huge malformations, removal of the mass is the best treatment for the patients and should be performed with caution because the tissues may bleed profusely. Therefore, patients may have long term survival after surgery.

## References

[1] Singh PP, Gupta N, Jain M (2001 ). Arteriovenous hemangioma involving submandibular salivary gland. Indian journal of otolaryngology and head and neck surgery : official publication of the Association of Otolaryngologists of India.

[2] Nour I, Abdel-Hady H, Nasef N, Shabaan AE (2014). A newborn with facial hemangioma and sternal defect. Journal of clinical neonatology.

[3] Ceisler EJ, Santos L, Blei F (2004). Periocular hemangiomas: what every physician should know. Pediatric dermatology.

[4] Drolet BA, Esterly NB, Frieden IJ (1999). Hemangiomas in children. The New England journal of medicine.

[5] Dumitriu D, Dudea S, Botar-Jid C, Baciut M, Baciut G (2011). Real-time sonoelastography of major salivary gland tumors. AJR American journal of roentgenology.

[6] Przewratil P, Sitkiewicz A, Wyka K, Andrzejewska E (2009 ). Serum levels of vascular endothelial growth factor and basic fibroblastic growth factor in children with hemangiomas and vascular malformations--preliminary report. Pediatric dermatology.

[7] Tisch M, Kraft K, Danz B, Maier H (2005). Cavernous hemangioma of the parotid gland in adults. Hno.

[8] Speight PM, Barrett AW (2002). Salivary gland tumours. Oral diseases.

[9] Khurana KK, Mortelliti AJ (2001). The role of fine-needle aspiration biopsy in the diagnosis and management of juvenile hemangioma of the parotid gland and cheek. Archives of pathology & laboratory medicine.

[10] Fanburg-Smith JC, Furlong MA, Childers EL (2003 ). Oral and salivary gland angiosarcoma: a clinicopathologic study of 29 cases. Modern pathology : an official journal of the United States and Canadian Academy of Pathology, Inc.

[11] Emsen IM (2008). Preoperative treatment of a parotid hemangioma with 100% ethyl alcohol. The Canadian journal of plastic surgery = Journal canadien de chirurgie plastique.

[12] Broeks IJ, Hermans DJ, Dassel AC, van der Vleuten CJ, van Beynum IM (2013). Propranolol treatment in life-threatening airway hemangiomas: a case series and review of literature. International journal of pediatric otorhinolaryngology.

[13] Weiss I, O TM, Lipari BA, Meyer L, Berenstein A, Waner M (201). Current treatment of parotid hemangiomas. The Laryngoscope.

[14] Morais P, Magina S, Mateus M, Trindade E, Jesus JM, Azevedo F (2011). Efficacy and safety of propranolol in the treatment of parotid hemangioma. Cutaneous and ocular toxicology.

[15] Mirbehbahani N, Rashidbaghan A (2014). Treatment process for capillary hemangioma. Iranian journal of pediatric hematology and oncology.

